# Karyotype structure and NOR activity in Brazilian *Smilax* Linnaeus, 1753 species (Smilacaceae)

**DOI:** 10.3897/CompCytogen.v13i3.35775

**Published:** 2019-08-22

**Authors:** Daniel Pizzaia, Vanessa M. Oliveira-Maekawa, Aline R. Martins, Mateus Mondin, Margarida L.R. Aguiar-Perecin

**Affiliations:** 1 Department of Genetics, Luiz de Queiroz College of Agriculture, ESALQ, University of São Paulo, Avenida Pádua Dias, 11, 13418-900 Piracicaba, SP, Brazil; 2 Department of Plant Biology, The University of Campinas, UNICAMP, Barão Geraldo, 13083-970, Campinas, SP, Brazil; 3 Department of Biological Sciences, Luiz de Queiroz College of Agriculture, ESALQ, University of São Paulo, Avenida Pádua Dias,11 13418-900, Piracicaba, SP, Brazil

**Keywords:** *
Smilax
*, karyotype, chromosomal evolution, nucleolus organizer region (NOR), Silver-staining, FISH, 45S rDNA

## Abstract

The genus *Smilax* Linnaeus, 1753 (Smilacaceae) is a large genus of dioecious plants distributed in tropical, subtropical and temperate regions. Some *Smilax* species have medicinal importance and their identification is important for the control of raw material used in the manufacture of phytotherapeutical products. The karyotypes of seven Brazilian *Smilax* species were investigated. Mitotic metaphases of roots from young plants were analysed in Feulgen-stained preparations. The karyotypes were asymmetric and modal with 2n = 2x = 32 chromosomes gradually decreasing in size. In *S. goyazana* A De Candolle & C De Candolle, 1878, a polyploid species, 2n = 4x = 64. In all the species, the large and medium-sized chromosomes were subtelocentric and submetacentric and the small chromosomes were submetacentric or metacentric. Their karyotypes were quite similar, with differences in the arm ratio of some chromosomes. *S. fluminensis* Steudel, 1841 differed from the other species by having a large metacentric chromosome 1. These findings suggest that evolution occurred without drastic changes in the chromosomal structure in the species analyzed. Terminal secondary constrictions were visualized on the short arm of some chromosomes, but they were detected only in one homologue of each pair. Due to the terminal location and the degree of chromosome condensation, secondary constrictions were not visualized in some species. The nucleolus organizer regions (NORs) were mapped by silver-staining and fluorescent *in situ* hybridization (FISH) in *S. rufescens* Grisebach, 1842 and *S. fluminensis*. Silver-staining and FISH signals were colocalized on the short arms of six chromosomes in *S. rufescens* and four chromosomes in *S. fluminensis*. In FISH preparations, one of the largest chromosomes had the secondary constrictions highly decondensed in some cells. This finding and the heteromorphism observed in Feulgen-stained chromosomes suggest that differential rRNA gene expression between homologous rDNA loci can occur in some cells, resulting in different degrees of ribosomal chromatin decondensation. The presence of a heteromorphic chromosome pair in *S. rufescens*, *S. polyantha* Grisebach, 1842 and *S. goyazana* suggests a chromosomal sex determination in these dioecious species.

## Introduction

The genus *Smilax* Linnaeus, 1753 (Smilacaceae) is a large genus of dioecious plants distributed in tropical, subtropical and temperate regions. The genus has approximately 300 species, and their classification has been controversial (see Mangaly 1968). This genus had been assigned to the family Liliaceae, but for the past 20 to 30 years, botanists have accepted Smilacaceae as a distinct family belonging to the order Liliales according to APG III (The Angiosperm Phylogeny Group 2009). The genus *Smilax* comprises the largest number of species within the Smilacaceae family, of which 32 species occur in Brazil (Andreata 1995, 2009).

Some *Smilax* species have medicinal importance. Roots have been used as anti-syphilitic, anti-inflammatory and antimicrobial remedies or as antioxidant agents (Andreata 1995, de Souza et al. 2004, Cox et al. 2005). The unequivocal characterization of *Smilax* species with potential medicinal applications is highly important, but some problems in the taxonomic identification of some species have been reported. The genus has high variability in morphology, and morphological features were described for species in Brazil (Andreata 2009), North America (Mangaly 1968) and Asia (Koyama 1960, Fu et al. 1995). Leaf morphology has been emphasized as an important feature for species identification by Mangaly (1968) and Andreata (2009). The possible evolution of inflorescences in *Smilax* and *Heterosmilax* Kunth, 1850 was considered (Kong et al. 2007). Reports on the morphoanatomy of vegetative organs (Martins and Appezzato-da-Gloria 2006, Martins et al. 2010, 2012) and molecular phylogeny (Sun al. 2015) have also contributed to species systematic.

The characterization of karyotypes in higher plants has evolutionary and taxonomic significance. Some studies on *Smilax* cytogenetics have reported chromosome numbers and karyotype characterization. Chromosome numbers of n = 16 were described for most species, but n = 13 and n = 15 were also recorded (Speese 1939, Mangaly 1968, Mehra and Sachdeva 1976, Vijayavalli and Mathew 1989, Fu and Hong 1990, Fu et al. 1993, 1995, Huang et al. 1997, Kong et al., 2007, Pizzaia et al. 2013; Sun et al. 2015). Some polyploids (n= 32, 48 and 64) have been found in Asian species (Vijayavalli and Mathew 1989, Fu and Hong 1990, Fu et al. 1993, 1995, Huang et al. 1997, Kong et al. 2007, Sun et al. 2015). The karyotypes of the species analyzed were asymmetric and modal, with most chromosomes being submetacentric and subtelocentric and all of them gradually decreasing in size (Vijayavalli and Mathew 1989, Fu and Hong 1990, Fu et al. 1993, 1995, Kong et al. 2007, Pizzaia et al. 2013). *Smilax* species are dioecious and heteromorphic chromosomes have been detected in some species, and are thought to be sexual chromosomes (Mangaly 1968, Vijayavalli and Mathew 1989, Fu et al.1995, Pizzaia et al. 2013). Secondary constrictions and satellites were detected in few species (Vijayavalli and Mathew 1989, Fu et al. 1995 and Pizzaia et al. 2013).

Pizzaia et al. (2013) described for the first time the nucleolus organizer regions of a *Smilax* species (*S. rufescens*), which were mapped by silver staining (Ag-NOR) and fluorescent *in situ* hybridization (FISH) of 45S rDNA probes. Silver signals colocalized with rDNA sites were observed on the short arms of six chromosomes.

In the present study, we investigated the karyotype characteristics of seven species of Brazilian species of *Smilax* using conventional techniques. We compared the positions of 45S rDNA sites of *S. rufescens* with the sites in *S. fluminensis*. Procedures to germinate wild-collected seeds to obtain plants providing roots for cytogenetic research were also developed. We aimed to analyze aspects of karyotype evolution in these species and to contribute to their taxonomic treatment.

## Material and methods

Seeds from wild plants collected from southern, southeastern, northeastern and western central Brazil were used (Table 1). The plants are dioecious, vines or herbaceous vines, or rarely, subshrubs or shrubs such as *S. goyazana* and *S. brasilienesis* Sprengel, 1825 (Andreata, 1995). The plants were identified by Dr. R.H.P Andreata (Santa Ursula University, Brazil) and were incorporated into the ESA herbarium (ESALQ, USP).

**Table 1.** Origin of collection, chromosome number, chromosome and haploid set length (µm) and ratio of the largest/smallest chromosomes of the *Smilax* species.

Species	Origin†	2n	Chromosome length		
			Range (µm)	Ratio (largest/ smallest)	Haploid set (µm)
*S. rufescens* Grisebach, 1842	Ilha do Cardoso (SP)	32	5.62–1.84	3.05	54.24
*S. fluminensis* Steudel, 1841	Uruana (GO)	32	6.41–1.33	4.82	43.47
	Itirapina (SP)	32	6.48–1.31	4.95	43.32
*S. polyantha* Grisebach, 1842	Botucatu (SP)	32	5.85–1.92	3.05	44.09
	Mogi Guaçu (SP)	32	5.36–1.94	2.80	41.03
*S. brasiliensis* Sprengel, 1825	Itapagipe (MG)	32	5.04–1.77	2.85	38.02
*S. campestris* Grisebach, 1842	Caçapava do Sul (RS)	32	6.20–1.95	3.18	43.44
*S. cissoides* Martius ex Grisebach, 1842	Feira de Santana (BA)	32	5.51–2.00	2.75	40.17
*S. goyazana* A. de Candolle & C de Candolle, 1878	Brasilia (DF)	64	4.85–1.61	3.01	79.25

†In brackets states of Brazil: SP (São Paulo), GO (Goias), MG (Minas Gerais), BA (Bahia), DF (Distrito Federal, Brasília).

Experiments to germinate seeds to obtain plants were evaluated as reported by Pizzaia et al. (2013). Briefly, the seeds were germinated in plastic boxes containing *Sphagnum* moss, at 27 °C. The seedlings were transferred to plastic pots containing vegetable soil + vermiculite and maintained under screenhouse conditions with the temperature varying from 20 °C to 32 °C. The sex of the young plants was not known.

Roots excised from young plants were pretreated with 8-hydroxyquinoline combined with cycloheximide, a protein synthesis inhibitor that induces chromosome condensation. Some treatments were evaluated, and in most cases, two treatments were used: combinations of 300 mg/L 8-hydroxyquinoline with 1.25 mg/L or 20 mg/L cycloheximide at 28 °C for 3 h and 2.5 h, respectively. The roots were then fixed in 3:1 ethanol:acetic acid and kept at 4 °C. The roots were Feulgen-stained as previously described (Bertão and Aguiar-Perecin 2002). For karyotype analysis, chromosomes of at least five metaphase spreads were measured. Chromosomes were identified according to their absolute length (µm), relative length (expressed as percentage of the haploid set), arm ratio (large arm/short arm) and chromosome types were designated according to Levan et al. (1964). The ratio of the largest chromosome/smallest chromosome was also described. The classification of the karyotypes according to their asymmetry (Stebbins, 1971) was adopted. The evaluation of the relative chromosome length in the tetraploid *S. goyazana* was carried out by estimating the percentage of the haploid set/2. The diploid-tetraploid comparisons were therefore based on one genome for the expression of the relative chromosome lengths.

Active NORs were detected in metaphase chromosomes of *S. rufescens* and *S. fluminensis*, by employing the silver-staining technique according to Stack et al. (1991) with minor modifications as previously reported (Pizzaia et al. 2013). Briefly, roots fixed in 3:1 etanol:acetic acid for 24 h were used to make squash preparations. The slides were heated for 2 h at 60 °C, incubated for 8 min in 2X SSC at the same temperature, washed with distilled water (3–5 min) and air-dried. Then, 50 µl 100% silver nitrate solution was added to the preparation, which was then covered with a nylon coverslip and incubated at 60 °C for 10 min on a Petri dish with moist filter paper. Coverslips were removed in tap water, and the slides were washed in distilled water, air-dried and mounted in Entellan (Merck, Germany).

Fluorescent *in situ* hybridization was used to detect 45S rDNA sites as previously described (Mondin et al. 2007). Metaphases of *S. rufescens* and *S. fluminensis* were investigated. Briefly, the probe used was the 9.1-kb maize 45S rDNA repeating unit labelled with biotin 14-dATP by nick translation (Bionick Labelling System, Invitrogen, USA). Biotin was detected with mouse anti-biotin followed by rabbit anti-mouse and swine anti-rabbit antibodies, both conjugated with TRITC (DAKO, Denmark). The probe (5 ng/µl) was added to the hybridization mixture and denatured by heating at 95 °C for 10 min. Hybridization was carried out at 37 °C for 20 h. Post-hybridization steps followed the protocol described by Mondin et al. (2007). The slides were counterstained with 1 µg/ml DAPI in Vectashield (Vector, USA).

All preparations were examined with a Zeiss Axiophot-2-epifluorescence microscope with appropriate filters. The images were acquired by a CCD camera using the IKAROS software to analyze the Feulgen-stained metaphases and the ISIS software for the FISH images (Meta-Systems, Germany). Silver-stained chromosomes were photographed using the Fujicolor Superia 100 film (Fuji Photo Film, Brazil). All images were processed with Adobe Photoshop 6.0.

## Results

### Karyotype anyses

The experiments to germinate seeds from wild plants were successful for obtaining plants and roots with high index of mitosis. The pretreatments used also provided metaphases suitable for karyotype analysis.

**Figure 1.**
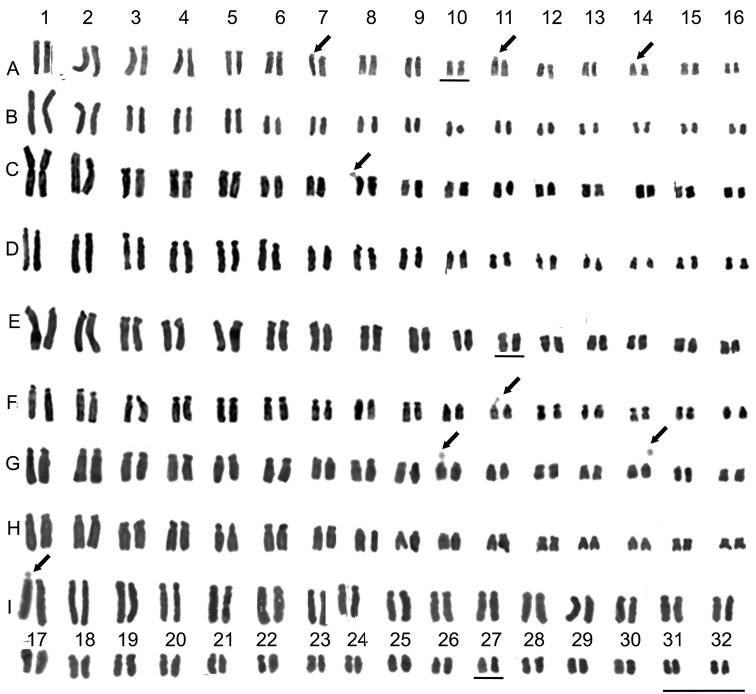
Karyotypes of *Smilax* species: Feulgen-stained metaphase chromosomes. **A***S. rufescens***B***S. fluminensis* (Uruana access) **C***S. fluminensis* (Itirapina access) **D***S. polyantha* (Botucatu access) **E***S. polyantha* (Mogi Guaçu access) **F***S. brasiliensis***G***S. campestris***H***S. cissoides***I***S. goyazana*. Arrows indicate secondary constrictions and satellites. Note the heteromorphic pair 10 in *S. rufescens*, pair 11 in *S. polyantha* (Mogi Guaçu) and pair 27 in *S. goyazana*. Scale bar: 10 µm.

All of the species analyzed had 2n = 2x = 32, except the tetraploid *S. goyazana* with 2n = 4x = 64. The karyotypes were asymmetric and modal with the chromosomes gradually decreasing in size, as shown in Fig. 1. The lengths of the haploid set and of the largest and smallest chromosomes (µm), as well as their ratio, are included in Table 1. The values of relative chromosome length, arm ratio, chromosome type, and Stebbins karyotype classification are given in Table 2. The karyotypes of these species are described for the first time, except for *S. rufescens* (Pizzaia et al. 2013), included here for comparison. The analysis of the karyotypes showed the following characteristics.

**Table 2.** Karyological data of Smilax species including chromosome relative length, arm ratio, karyotype type.

**Chromosomes**																	1	
**Species**																		
**Relative length (%)**	**1**	**2**	**3**	**4**	**5**	**6**	**7**	**8**	**9**	**10**	**11**	**12**	**13**	**14**	**15**	**16**		
*S. rufescens*	10.37	9.64	8.70	8.23	7.55	7.08	6.36	6.03	5.82	5.06/5.06	4.75	4.57	4.35	4.09	3.87	3.41		
*S. fluminensis* (Uruana)	15.08	11.32	9.25	8.84	8.26	6.50	5.87	5.59	5.11	4.81	4.03	4.04	3.95	3.77	3.53	3.33		
*S. fluminensis* (Itirapina)	15.79	10.87	8.67	8.35	8.16	7.00	6.31	5.96	5.43	5.04	4.26	4.24	4.09	4.01	3.77	3.13		
*S. polyantha* (Botucatu)	11.80	10.29	9.68	9.01	8.29	7.88	6.99	6.76	5.94	5.21	5.00	4.75	4.56	4.43	4.05	3.90		
*S. polyantha* (Mogi Guaçu)	10.52	9.71	8.86	8.15	7.90	6.95	6.85	6.07	5.95	5.59	4.91/491	4.37	4.34	3.88	3.06	3.02		
*S. brasiliensis*	11.82	10.50	10.00	9.24	8.74	8.17	7.61	6.94	6.44	5.41	5.19	4.89	4.89	4.80	4.65	4.17		
*S. campestris*	11.06	9.32	8.54	8.18	7.47	7.25	6.70	6.40	5.65	5.32	4.63	4.14	4.14	3.85	3.81	3.47		
*S. cissoides*	10.33	9.39	8.18	7.97	7.70	7.24	6.56	6.11	5.82	5.28	4.69	4.23	4.19	3.57	3.57	3.50		
*S. goyazana*	5.51	5.51	5.00	4.50	4.48	4.38	4.29	4.28	3.79	3.69	3.64	3.59	3.45	3.28	3.18	3.09		
**Chromosomes**																		
	**17**	**18**	**19**	**20**	**21**	**22**	**23**	**24**	**25**	**26**	**27**	**28**	**29**	**30**	**31**	**32**		
*S. goyazana* (cont)	2.94	2.85	2.85	2.73	2.69	2.17	2.09	2.05	1.97	1.94	1.89/1.89	1.89	1.85	1.77	1.71	1.67		
**Chromosomes**																		
**Arm ratio / Chromosome types**	**1**	**2**	**3**	**4**	**5**	**6**	**7**	**8**	**9**	**10**	**11**	**12**	**13**	**14**	**15**	**16**	**Karyotypes**	**Stebbins karyotype classification**
*S. rufescens*	5.28	4.13	4.46	3.41	3.28	3.00	3.30	2.56	2.82	2.17/1.67	2.15	1.99	1.92	1.84	1.53	1.24	7st+6.5sm+2.5m	3B
	st	st	st	st	st	st	st	sm	sm	sm/m	sm	sm	sm	sm	m	m		
*S. fluminensis* (Uruana)	1.02	2.02	3.17	2.91	3.03	4.00	2.50	2.70	2.98	2.55	2.31	3.00	1.77	1.49	1.70	1.85	4st+10sm+2m	3C
	m	sm	st	sm	st	st	sm	sm	sm	sm	sm	st	sm	m	sm	sm		
*S. fluminensis* (Itirapina)	1.07	2.08	3.00	2.68	3.03	3.03	1.60	3.0	2.24	2.49	2.03	3.00	1.84	1.49	1.56	1.78	5st+7sm+4m	3C
	m	sm	st	sm	st	st	m	st	sm	sm	sm	st	sm	m	m	sm		
*S. polyantha* (Botucatu)	6.10	6.00	5.00	4.00	4.00	3.8	3.10	3.00	2.11	2.43	1.59	3.00	2.50	1.45	1.35	1.24	9st+3sm+4m	3B
	st	st	st	st	st	st	st	st	sm	sm	m	st	sm	m	m	m		
*S. polyantha* (Mogi Guaçu)	6.00	6.00	4.00	3.50	3.50	3.17	3.17	3.00	3.00	3.00	2.00/3.00	2.04	2.00	1.60	1.50	1.24	10.5st+2.5sm+3m	3B
	st	st	st	st	st	st	st	st	st	st	sm/st	sm	sm	m	m	m		
*S. brasiliensis*	6.00	5.10	4.00	3.90	3.38	3.17	3.00	2.72	2.54	2.74	3.00	1.89	2.00	1.78	1.47	1.50	8st+6sm+2m	3B
	st	st	st	st	st	st	st	sm	sm	sm	st	sm	sm	sm	m	m		
*S. campestris*	4.51	4.1	3.23	2.94	3.18	2.27	3.00	2.7	1.99	2.17	1.5	1.50	1.38	1.5	1.62	1.4	5st+5sm+6m	3B
	st	st	st	sm	st	sm	st	sm	sm	sm	m	m	m	m	m	m		
*S. cissoides*	4.60	4.54	3.00	3.82	2.79	3.44	2.4	3.00	3.00	2.00	2.00	1.70	1.87	2.00	1.49	1.50	7st+7sm+2m	3B
	st	st	st	st	sm	st	sm	st	st	sm	sm	sm	sm	sm	m	m		
*S. goyazana*	8.00	7.5	5.75	4.35	4.89	5.01	4.16	4.00	3.00	3.17	3.00	2.7	3.44	3.10	3.03	3.00	2t+15.5st+ 8.5sm+6m	3B
	t	t	st	st	st	st	st	st	st	st	st	sm	st	st	st	st		
**Chromosomes**																		
	**17**	**18**	**19**	**20**	**21**	**22**	**23**	**24**	**25**	**26**	**27**	**28**	**29**	**30**	**31**	**32**		
*S. goyazana* (cont)	3.00	2.96	2.96	2.7	3.00	1.83	1.5	1.78	2.50	2.60	3.00/2.09	1.17	1.18	1.32	1.43	1.40		
	st	sm	sm	sm	st	sm	m	sm	sm	sm	st/sm	m	m	m	m	m		

t: telocentric, st: subtelocentric, sm: submetacentric, m: metacentric

### *S. rufescens* Grisebach, 1842

This species with 2n = 2x = 32 showed a karyotype with 7 pairs of st-type, 6 pairs of sm-type and 2 pairs of m-type chromosomes, and the heteromorphic pair 10 with sm-and m-type chromosomes with similar sizes, probably sex chromosomes. The size of the chromosomes varied from 1.84 to 5.62 µm with a largest/smallest ratio of 3.05 and the Stebbins karyotype classifications was 3B. The total haploid length was 54.24 µm. Secondary constrictions were detected on the short arms of chromosomes 7, 11 and 14 in some metaphases. These constrictions were visible only in one homologue of each chromosome pair. (Fig. 1A; Tables 1, 2). As it was unclear if satellites were visible, the detected structures were considered as secondary constrictions (Pizzaia et al. 2013).

### *S. fluminensis* Steudel, 1841

Specimens collected from Uruana (GO) and Itirapina (SP) were analyzed. Both specimens had 2n= 2x = 32. The karyotypes were quite similar with slight differences in the arm ratio of chromosomes 7, 8 and 15. The plants from Uruana showed a karyotype with 4 pairs of st-type, 10 pairs of sm-type and 2 pairs with m-type chromosomes. The size of the chromosomes varied from 1.33 to 6.41 µm with a largest/ smallest ratio of 4.82 and the Stebbins karyotype classification was 3C. The total haploid length was 43.47 µm. Secondary constrictions were not detected (Fig. 1B, Tables 1, 2). The plants from Itirapina (SP) showed a karyotype with 5 pairs of st-type, 7 pairs of sm-type and 4 pairs of m-type chromosomes. The size of the chromosomes varied from 1.31 to 6.48 µm with a largest/ smallest ratio of 4.95 and the Stebbins karyotype classification was 3C. The total haploid length was 43.42 µm. A satellite and secondary constriction were detected on the short arm of chromosome 8 and were visible only in one homologue of the chromosome pair (Fig. 1C, Tables 1, 2).

### *S. polyantha* Grisebach, 1842

Specimens collected from Botucatu (SP) and Mogi Guaçu (SP) were analyzed. Both had 2n = 2x = 32. The karyotypes were quite similar with slight differences in the arm ratio of chromosomes 9, 10, 11 and 12. The plants from Botucatu showed a karyotype with 9 pairs of st-type, 3 pairs of sm-type and 4 pairs of m-type chromosomes. The size of chromosomes varied from 1.92 to 5.85 µm with a largest/smallest ratio of 3.05 and the Stebbins karyotype classification was 3B. The total haploid length was 44.09. Secondary constrictions were not detected (Fig. 1D, Tables 1, 2). The plants from Mogi Guaçu (SP) showed a karyotype with 10 pairs of st-type, 2 pairs of sm-type and 3 pairs of m-type chromosomes and the heteromorphic pair 11 with sm-type and st-type chromosomes of similar sizes, probably sexual chromosomes. The size of the chromosomes varied from 1.94 to 5.36 µm with a largest/smallest ratio of 2.80 and the Stebbins karyotype classification was 3B. The total haploid length was 41.03 µm. Secondary constrictions were not detected (Fig. 1E, Tables 1, 2).

### *S. brasiliensis* Sprengel, 1825

This species with 2n = 2x = 32 showed a karyotype with 8 pairs of st-type, 6 pairs of sm-type and 2 pairs of m-type chromosomes. The size of chromosomes varied from 1.77 to 5.04 µm with a largest/smallest ratio of 2.85 and the Stebbins karyotype classification was 3B. The total haploid length was 38.02 µm. A satellite and secondary constriction were observed on chromosome 11 and they were visible only in one homologue of the chromosome pair (Fig. 1F, Tables 1, 2).

### *S. campestris* Grisebach, 1842

This species with 2n = 2x = 32 showed a karyotype with 5 pairs of st-type, 5 pairs of sm-type and 6 pairs of m-type chromosomes. The size of chromosomes varied from 1.95 to 6.20 µm with a largest/smallest ratio of 3.18 and the Stebbins karyotype classification was 3B. The total haploid length was 43.44 µm. Satellites and secondary constrictions were detected on the chromosomes 10 and 14, visible only in one homologue of each pair (Fig. 1G, Tables 1, 2).

### *S. cissoides* Martius ex Grisebach, 1842

This species with 2n = 2x = 32 showed a karyotype with 7 pairs of st-type, 7 pairs of sm-type and 2 pairs of m-type chromosomes. The size of chromosomes varied from 2.00 to 5.51 µm with a largest/smallest ratio of 2.75 and the Stebbins karyotype classification was 3B. The total haploid length was 40.17 µm. Satellites were not detected (Fig. 1H, Tables 1, 2).

### *S. goyazana* A de Candolle & C de Candolle, 1878

This polyploid species with 2n = 4x = 64 showed a karyotype with 2 pairs of t-type, 15 pairs with st-type, 8 pairs with sm-type, 6 pairs with m-type chromosomes and the heteromorphic pair 27 with st-type and sm-type chromosomes with similar sizes. The size of chromosomes varied from 1.61 µm to 4.85 with a largest/smallest ratio of 3.01 and the Stebbins karyotype classification was 3B. The total haploid length was 79.25 µm. A satellite and secondary constriction were detected on the largest and t-type chromosome 1, and it was visible only in one of the homologues (Fig. 1I, Tables 1, 2).

### NOR-regions visualized by silver staining and FISH

**Figure 2.**
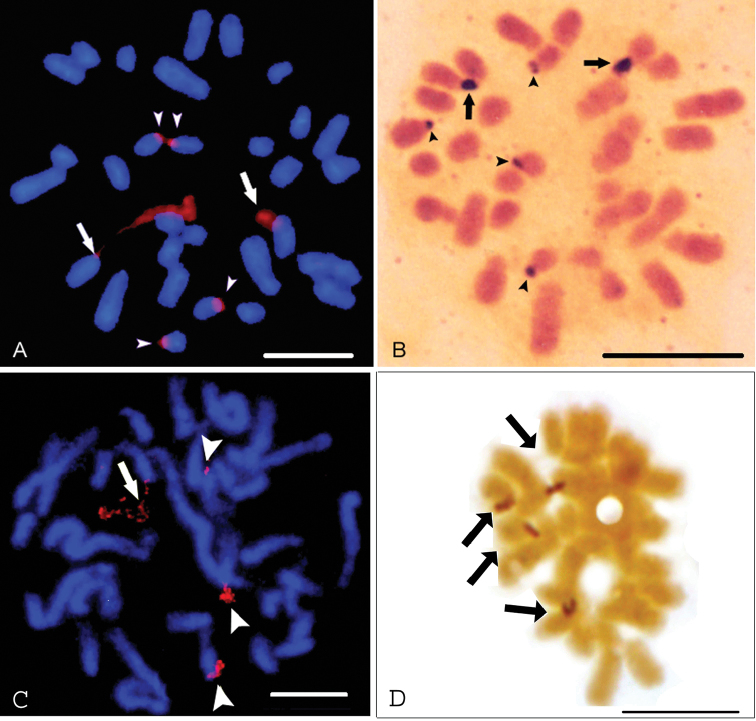
FISH signals of 45S rDNA (red) (**A, C**), silver staining (**B, D**) in *S. rufescens* (**A, B**) and *S. fluminensis* (**C, D**). Arrows in **A** and **C** indicate distended secondary constrictions, and arrowheads show condensed rDNA sites. Arrows in **B** and **D** indicate larger silver signals and arrowheads in B shows small sites. Scale bar: 10 µm.

As previously reported (Pizzaia et al. 2013), *in situ* hybridization detected sites of 45S rDNA in six chromosomes of *S. rufescens*. A larger pair of medium-sized chromosomes showed distended secondary constrictions with different lengths between the homologues (Fig. 2A). This pair must correspond to chromosome 7 shown in Fig. 1A. Metaphases with both homologues displaying similar lengths of stretched secondary constrictions were also visualized (not shown). Two pairs of the smallest chromosomes showed minor sites of ribosomal DNA, and they must correspond to pairs 11 and 14, as shown in Fig. 1A. Secondary constrictions were not detected in these small chromosomes in FISH preparations.

In the silver-stained metaphases of *S. rufescens*, six chromosomes showed positive signals on the termini of the short arms (Fig. 2B), thereby giving evidence that the six sites of rDNA were active. Two chromosomes had larger signals, and minor silver-stained sites were observed in four chromosomes, as previously shown (Pizzaia et al. 2013).

In *S. fluminensis*, only four chromosomes showed 45S rDNA sites. In Fig. 2C, which illustrates a prometaphase, a highly distended secondary constriction is seen in one medium-sized chromosome that must correspond to the satellited chromosome 8 visualized in Fig. 1C. The other two chromosomes show secondary constrictions that are less distended. A small signal is observed in a smaller chromosome. One metaphase with two chromosomes showing highly distended secondary constrictions was also observed (not shown).

In the silver-stained metaphases of *S. fluminensis*, four chromosomes showed positive signals on the termini of short arms (Fig. 2D). The signals had the same size.

## Discussion

### Karyotype analyses

All of the diploid species studied here had 2n = 2x = 32, except the polyploid *S. goyazana* with 2n = 4x= 64. Most diploid species from East Asia and India also have 2n = 2x = 32 (Vijayavalli and Mathew 1989, Fu and Hong 1990, Fu et al. 1993, 1995, Huang et al. 1997, Kong et al. 2007, Sun et al. 2015), while a survey of species from North America (USA) found most of the species with 2n = 2x = 26 and one species with 2n = 2x = 30 (Mangaly 1968). The chromosome number in polyploids is variable (2n = 64, 96, 128 (Vijayavalli and Mathew 1989, Fu and Hong 1990, Fu et al. 1993, 1995, Kong et al. 2007), all based on n = 16. In the present study, the tetraploid *S. goyazana* has 2n = 4x =64. In some species, populations with variable numbers of chromosomes were described, such as the *S. china* Linnaeus, 1753 complex, in which diploids (2n = 30, 32) and polyploids (2n = 64, 96) were described (see Sun et al. 2015). The species investigated in our study have n = 16, thereby giving additional evidence that this must be the basic number in the genus *Smilax*. The chromosome number of polyploids is also derived from the basic number x = 16.

The karyotypes of the species analyzed were asymmetric, and the absolute size of the chromosomes was rather similar, gradually decreasing in size, except in *S. fluminensis*, in which the metacentric chromosome 1 was larger than in the other species. This finding was clearly emphasized with the evaluation of the ratio between the largest and smallest chromosomes that varied from 2.75 to 3.18 in most species compared with the values of *S. fluminensis* that were larger (4.82–4.95). The relative chromosome lengths were also quite similar with clear differences concerning the relative lengths of chromosome 1 in most species (10.33 to 11.82) compared with *S. fluminensis* (15.08–15.79). In a general sense, these data were similar to those reported for species from East Asia (Fu and Hong 1990, Fu et al. 1993, 1995, Kong et al. 2007). From this finding, we can conclude that chromosomal evolution in *Smilax* was not accompanied by large modifications in chromosome and haploid set size, as well as large chromosomal rearrangements in the species with 2n = 32.

The presence of a large metacentric chromosome 1 in the karyotype of *S. fluminensis* is a special feature that is unusual in *Smilax* species. Fu et al. (1995) described the karyotype of *S. mirtillus* A de Candolle & C de Candlolle, 1878 with a second heteromorphic pair showing m- and sm-chromosomes as a special karyotype. Additionally, a heteromorphic subtelocentric pair 1 was found in two *Smilax* species by Fu et al. (1995). A size heteromorphism of the homologues of the satellited pair 7 was detected in male plants of *S. aspera* Linnaeus, 1753 by Vijayavalli and Mathew (1989). These authors assumed an XY (male) and XX (female) type of chromosome sex complex for this species. In our study, in *S. rufescens*, pair 10 was heteromorphic for their centromere positions, and the chromosomes were not NOR-bearing chromosomes. In *S. polyantha* (from Mogi-Guaçu) and *S. goyazana* we also detected heteromorphism related to centromere position in pairs 11 and 27, respectively. As we used young plants, we had no information on their sex, therefore we can speculate only that these heteromorphic pairs are sexual chromosomes. These findings give evidence that differentiated sexual chromosomes must be a characteristic of *Smilax* karyotypes, however more analyses are needed for deeper conclusions.

In general, the species analyzed had asymmetric karyotypes with chromosomes gradually decreasing in size and showing variability in centromere position. The classification of the karyotypes according to Stebbins (1971) showed that all the karyotypes were classified as 3B, except *S. fluminensis* that was 3C. Interestingly, most karyotypes of species 2n=32 from China, analyzed by Fu et al. (1993, 1995), also belong to class 3B, with few of them classified as 3C. In the present investigation, in most species, the largest and medium-sized chromosomes were subtelocentric and submetacentric. The smallest chromosomes were submetacentric and metacentric. For instance, *S. polyantha* (from Mogi Guaçu) had the highest number of subtelocentric chromosomes among the largest ones, and in *S. campestris*, the highest number of submetacentric and metacentric types was detected among the medium-sized and smallest chromosomes. The features observed suggest that during the evolution of these species, slight alterations occurred in the position of the centromeres, probably due to different accumulation of repetitive DNA in chromosome arms, as discussed below. Only *S. fluminensis* with a large metacentric chromosome 1 suffered a different type of chromosome rearrangement.

The genus *Smilax* has been assigned to the family Smilacaceae and to the order Liliales sensu APG III thus, it is a sister family of Liliaceae. Peruzzi et al. (2009) considered that the ancestral basic number for Liliaceae is x=8, based on the frequency of counts 2n=32 in *Smilax* and cytological data suggesting this genus to be paleopolyploid, according to Vijayavalli and Mathew (1989). Peruzzi et al (2009) reported that *Smilax* species have small mean genome size (9.16 pg) compared with some Liliaceae, such as the genera *Streptopus* Michaux, 1803 (3.43 pg), and *Prosartes* D Don, 1830 (5.08 pg), which also have a small mean genome size in contrast with the tribe Lilieae, in which genera with large genomes were found: *Lilium* Linnaeus, 1753 (56.31 pg) *Fritillaria* Linnaeus, 1753 (44.49 pg), *Cardiocrinum* Lindley, 1846 (36.18 pg) and *Notholirion* Boissier, 1889 (27.82 pg).

Peruzzi et al. (2009) suggested that the ancestral Liliaceae species would have a small genome size and that evolution occurred in the direction of increasing size. In general, it is well known that in plants there is a positive correlation between genome size and the amount of repetitive DNA for both *in tandem* and dispersed repeats. For instance, in maize, the evolution from an ancestral plant occurred with an increase in the content of repetitive DNA. Expressive variation in genome size was observed among maize varieties (Laurie and Bennett 1985). The analysis of the inbred line B73 (reference genome) showed that 85% of the genome was composed of transposable elements, of which 75% belonged to LTR retrotransposon families (Schnable et al. 2009). The arm ratios of the knobless chromosomes 2 and 4 of the inbred line KYS compared with their homologues in the tropical JD lines were significantly different, possibly due to differences in their content of repetitive DNA (Mondin et al. 2014). From this scenario, we could infer that the differences in arm ratios observed among *Smilax* species analyzed in this study are due to different contents of repetitive DNA in their chromosome arms.

Significant differences in karyotype asymmetry are apparent within Liliales, in which two different aspects can be observed, such as variation in chromosome length and variation in centromere position. Variation in chromosome lengths such as the observed in *Smilax*, was observed in some Liliaceae genera with small genome sizes such as *Streptopus* and *Prosartes*, while in some genera, such as *Lilium* and *Fritillaria*, the asymmetry is mainly due to variation in the position of centromeres (two large metacentric chromosomes and subtelocentric and telocentric chromosomes with rather similar lengths (see Peruzzi et al. 2009).

Secondary constrictions were not described for the karyotypes of most *Smilax* species reported in the literature. Vijayavalli and Mathew (1989) detected satellites on the short arm of subtelocentric chromosomes in three species (*S. aspera*, *S. bracteata* Presl, 1827 and *S. zelanica* Linnaeus, 1753) and a secondary constriction on the long arm of chromosome 1 in *S. wightii* A de Candolle & C de Candolle, 1753 but did not detect secondary constrictions in the karyotypes of the cytotypes of the polyploid *S. ovalifolia* Roxburgh, 1832. Fu et al. (1995) reported the presence of secondary constrictions only on the long arm of two subtelocentric pairs (second and third) in *S. corbularia* Kunth, 1850 in a study involving nine species of *Smilax* and three species of *Heterosmilax*. In our study, we detected secondary constrictions on the short arms of subtelocentric chromosomes in *S. rufescens*, *S. fluminensis*, *S. brasiliensis*, *S. campestris* and the polyploid *S. goyazana* in conventionally stained preparations. All of these constrictions were located on medium-sized and small chromosomes, except for *S. goyazana* which has a large chromosome 1 bearing a secondary constriction and satellite. As discussed below, the 45S rDNA is located on the termini of the NOR-chromosome short arms thus, due to the degree of chromosome condensation, secondary constrictions might not be visualized in conventionally stained chromosomes.

### NOR regions

In the present study, *in situ* hybridization of 45S rDNA probes showed six sites of ribosomal DNA in *S. rufescens* and four sites in *S. fluminensis*. In addition to differing from *S. rufescens* and the other species analyzed by the presence of a large metacentric chromosome 1, *S. fluminensis* has a different number of ribosomal DNA sites. The number of positive silver-staining signals was correlated with the FISH signals in both species. Terminal secondary constrictions were observed in three chromosome pairs of *S. rufescens*. As it was unclear if satellites were visible, the structures visualized were considered terminal secondary constrictions. In more condensed metaphases, only two chromosomes displayed secondary constrictions (Pizzaia et al. 2013). In the Itirapina access of *S. fluminensis*, only one chromosome exhibited a secondary constriction, and a satellite was observed. In Fig. 1C, the formation of a satellite is more conspicuous. Therefore, in the case of *S. fluminensis*, we find that due to the terminal position of the NOR region and the degree of chromosome condensation, secondary constrictions could not be detected in all chromosomes.

Secondary constrictions have been considered to be the organization pattern of active ribosomal chromatin on metaphase chromosomes. Silver-staining on secondary constriction allows the visualization of ribosomal genes that were transcribed in the previous interphase. It has been shown that silver binds to proteins that are components of the transcription machinery and remain at NOR regions throughout metaphase and anaphase (see revision in Caperta et al. 2002). The organization pattern of the rDNA site visualized by FISH in one metaphase chromosome pair in species such as *Secale cereale* Linnaeus, 1753 (Caperta et al 2002; 2007) and *Zea mays* Linnaeus, 1753 (Kato et al. 2004, Mondin et al. 2014) is a distended secondary constriction and a condensed block of proximal ribosomal chromatin, which is transcriptionally inactive. As the NOR region is localized on a subterminal position of the short chromosome arm in these species, a satellite stained by DAPI is visualized. In both *S. rufescens* and *S. fluminensis*, a condensed chromatin block was not visualized in the chromosome presenting a distended secondary constriction, and neither a DAPI-stained satellite was detected. This last observation provides evidence that the rDNA sites are localized at chromosome termini. Therefore, we suppose that the satellite observed in the Feulgen-stained metaphase of *S. fluminensis* shown in Fig. 1C would be a structure containing rDNA, as in species of *Passiflora* Linnaeus, 1753 in which ribosomal DNA was observed on secondary constrictions and satellites (Cuco et al. 2005).

Details on the structure and function of the NOR chromosomes, as well as the quantification of observed events were not the scope of this study, but the detection of some features allows some discussion. The presence of positive-silver staining signals corresponding with the number of rDNA sites revealed by FISH showed that all these loci were active in both species analyzed. However, in some Feulgen-stained metaphases of *S. rufescens* only two chromosomes bearing secondary constriction were observed (Pizzaia et al. 2013), whereas in the metaphase of *S. fluminensis* seen in Fig. 1B, secondary constrictions were not detected, and in Fig. 1C, only one was observed. As mentioned above, these findings are a consequence of the terminal position of the rDNA loci and the degree of chromosome condensation not allowing the visualization of the secondary constrictions. Additionally, in the FISH preparation of *S. rufescens* shown in Fig. 2A, one largest NOR chromosome has a secondary constriction that is highly distended in comparison with its homologous. This chromosome would be more active than its homologue. This heteromorphism of the secondary constriction was observed in FISH preparations of *S. cereale*, giving evidence of differential expression of homologous rDNA loci (Caperta et al. 2002; 2007). This behavior was also supported by the observation of differences in the size of the silver-staining signals. In an experiment carried out to evaluate this event, metaphases showing heteromorphic secondary constriction were more frequent than the homomorphic ones, and the rDNA loci on homologous chromosomes had equivalent numbers of ribosomal cistrons (Caperta et al. 2002).

In the present study, heteromorphic secondary constrictions were detected in FISH preparations of *S. rufescens* and *S. fluminensis*, but metaphases with both homologues of the largest NOR chromosome pair displaying distended secondary constrictions were also observed (not shown).Therefore, we conclude that in these species, differential expression of rDNA loci occurs only in some cells. The observation of homologous NOR chromosomes showing differences in the presence of secondary constrictions in Feulgen-stained metaphases of *S. rufescens*, *S. fluminensis* and also in *S. campestris*, *S. cissoides* and *S. goyazana* suggests that differential expression of rDNA loci is frequent in the genus *Smilax*.

Interestingly, the treatment of roots of *S. cereale* with the methyltransferase inhibitor 5-azacytidine (5-AC) resulted in an increase of rRNA gene transcription and then in a reduction in the number of cells showing a significant difference in the size of silver-stained domains in the two NORs (Caperta et al. 2007). The authors concluded that ribosomal gene silencing was controlled by DNA methylation and that rRNA gene transcription, silver-staining and NOR chromatin decondensation were interrelated in *S. cereale*. Fig. 2B shows two apparently non-homologous chromosomes of *S. rufescens* with larger silver-stained domains, suggesting the occurrence of differential rRNA expression. Fig. 2D illustrates NOR chromosomes of *S. fluminensis* with silver-stained NORs of similar sizes. These observations provide evidence that differential rRNA gene expression, as well as equal expression occur in these species.

The differential expression between homologous NOR chromatin is a different phenomenon in relation to nucleolar dominance. Nucleolar dominance has been characterized as an epigenetic phenomenon that occurs in plant allopolyploids and hybrids, in which only one ancestral set of ribosomal genes retains the ability to organize the nucleolus, while the rDNA loci derived from the other progenitor are silenced. For instance, in *Atropa belladonna* Linnaeus, 1753 derived from a tetraploid and a diploid ancestor species, only four out of six rDNA sites are transcriptionally active, as revealed by silver-staining (Volkov et al. 2017). In *Quercus robur* Linnaeus, 1753, two rDNA loci were observed, NOR-1 and NOR-2 (Bockor et al. 2014). Only NOR-1 showed decondensed chromatin in FISH preparations and positive silver signals. In interphases, NOR-2 was condensed and located away from the nucleolus, while the major locus (NOR-1) was associated with the nucleolus and exhibited different degrees of condensation. Treatment with 5-azacytidine increased the total level of RNA transcripts and decreased the degree of DNA methylation at NOR-2 site however, the chromatin condensation of this locus was not affected, suggesting that NOR-2 has lost the function of rRNA synthesis and nucleolus organization.

## Conclusions

The karyotypes of seven Brazilian *Smilax* species investigated were asymmetric and modal with 2n = 2x = 32 chromosomes gradually decreasing in size. In *S. goyazana*, a polyploid species, 2n = 4x = 64. In all the species, the large and medium-sized chromosomes were subtelocentric and submetacentric and the small chromosomes were submetacentric or metacentric. Their karyotypes were quite similar, with slight differences in the arm ratio of some chromosomes and belong to class 2B according with Stebbins classification (1971). *S. fluminensis* differed from the other species by having a large metacentric chromosome 1 and belonging to class 3C. These findings suggest that evolution occurred without drastic changes in karyotype structure in the species analyzed, except *S. fluminenesis*. Terminal secondary constrictions were visualized on the short arm of some chromosomes, but they were detected only in one homologue of each pair. Due to the terminal location and the degree of condensation of the chromosomes, secondary constrictions were not visualized in some species. In *S. rufescens* and *S. fluminensis* all the rDNA loci were active as demonstrated by silver-staining signals colocalized with the FISH signals. We concluded that differential expression of rDNA loci occurs in these species based on the observation of a distended secondary constriction in the largest NOR chromosome visualized in FISH preparations of some cells in both species. Distended secondary constrictions were not observed in the smallest chromosomes, probably due to their small size and the degree of metaphase condensation. In this connection, it is interesting to note that the absence of secondary constriction on an active locus was observed in *Crotalaria juncea* Linnaeus, 1753, in which two silver-stained rDNA loci were observed, that is, one major locus showing secondary constriction and one minor locus in which a secondary constriction was not detected (Mondin et al. 2007). The largest silver signals were observed in two chromosomes in *S. rufescens*, while signals with the same size were observed in *S. fluminensis*, demonstrating, in this species, that differential expression of rRNA genes does not occur in some cells. Additionally, the observation of homologous NOR chromosomes with differences in the presence of secondary constrictions in Feulgen-stained metaphases of *S. rufescens*, *S. fluminensis*, and in *S. campestris*, *S. cissoides* and *S. goyazana* suggests that *Smilax* is an interesting genus for further studies on the activity of the NOR sites.
